# Molecular detection of cattle *Sarcocystis* spp. in North-West Italy highlights their association with bovine eosinophilic myositis

**DOI:** 10.1186/s13071-021-04722-5

**Published:** 2021-04-23

**Authors:** Selene Rubiola, Tiziana Civera, Felice Panebianco, Davide Vercellino, Francesco Chiesa

**Affiliations:** 1 Department of Veterinary Sciences, University of Turin, Largo Paolo Braccini 2, 10095 Grugliasco, TO Italy; 2 Veterinary Service (A.S.L. AT), 14100 Asti, AT Italy

**Keywords:** *Sarcocystis* spp., Bovine eosinophilic myositis (BEM), *Cox1* (COI) gene, 18S rDNA gene, Cattle, Molecular identification, *Sarcocystis hominis*, Zoonotic

## Abstract

**Background:**

Cattle are intermediate hosts of six *Sarcocystis* species, among which *Sarcocystis hominis* and *Sarcocystis heydorni* can infect humans through the consumption of raw or undercooked meat. In addition to the zoonotic potential, there is increasing interest in these protozoa because of the evidence supporting the role of *Sarcocystis* spp. in the occurrence of bovine eosinophilic myositis (BEM), a specific inflammatory myopathy which leads to carcass condemnation and considerable economic losses. Actually, all the prevalence studies carried out on cattle in Italy have been based on either morphological or 18S rDNA-based molecular techniques, most likely leading to misidentification of closely related species. Therefore, there is a strong need for new data on the prevalence of the different *Sarcocystis* spp. in cattle in Italy and their association with bovine eosinophilic myositis.

**Methods:**

To reach our aim, individual striated muscle samples from BEM condemned carcasses (*N* = 54) and diaphragm muscle samples from randomly sampled carcasses (*N* = 59) were obtained from Northwest Italy slaughterhouses. Genomic DNA was extracted and analyzed by multiplex-PCR targeting 18S rDNA and *cox1* genes. PCR products amplified using the genus-specific primer set in absence of the specific fragment for *S. hirsuta*, *S. cruzi*, *S. hominis* or *S. bovifelis* were sequenced to achieve species identification.

**Results:**

*Sarcocystis* DNA was detected in 67.8% of the samples from slaughter cattle and in 90.7% of the samples from BEM condemned carcasses. *S. cruzi* was identified as the most prevalent species in slaughter cattle (61%), followed by *S. bovifelis* (10.2%), *S. hominis* (8.5%) and *S. hirsuta* (1.7%). Notably, among the different *Sarcocystis* spp. detected, the presence of *S. bovifelis* and *S. hominis* was significantly higher in samples isolated from BEM condemned carcasses (46.3% and 40.7% respectively), while there was no statistically significant difference between the presence of *S. cruzi* or *S. hirsuta* in BEM condemned carcasses (42.6% and 1.8%, respectively) and randomly sampled carcasses. Furthermore, DNA sequence analysis revealed the presence of a putative new species in two carcasses.

**Conclusions:**

Our study contributes to updating the data on the prevalence of the different *Sarcocystis* spp. in cattle in Italy, highlighting the presence of three *Sarcocystis* spp., *S. cruzi*, *S. hominis* and *S. bovifelis*, in BEM lesions and allowing us to speculate on the possible role of *S. hominis* and *S. bovifelis* as the major sarcosporidian species involved in bovine eosinophilic myositis.

**Graphic Abstract:**

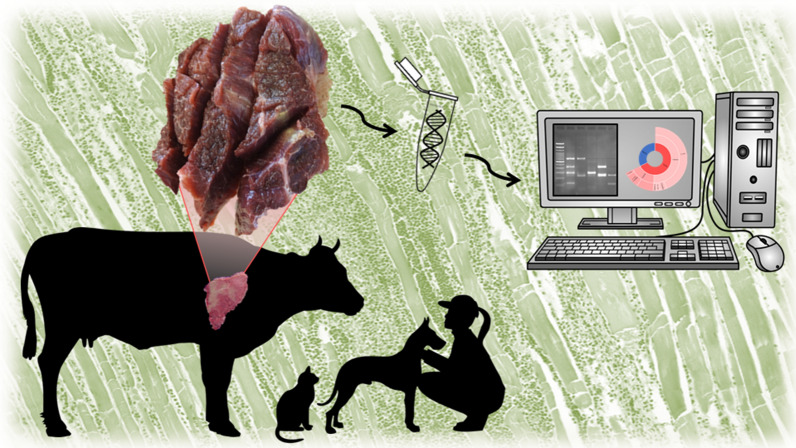

**Supplementary Information:**

The online version contains supplementary material available at 10.1186/s13071-021-04722-5.

## Background

*Sarcocystis* species are protozoan parasites belonging to the phylum Apicomplexa. The genus *Sarcocystis* consists of more than 200 species characterized by a worldwide distribution, three of which—*S. hominis*, *S. heydorni* and *S. suihominis*—are known to use humans as definitive hosts [1]. These latter become infected via the ingestion of cysts in muscular tissues, while the intermediate hosts acquire infection by ingesting oocysts and sporocysts in feed or water. Humans can develop two different clinical forms of sarcocystosis: an intestinal form, caused by *S. hominis*, *S. heydorni* and *S. suihominis*, and a muscular form, caused by *S. nesbitti*, the only *Sarcocystis* sp. that uses humans as intermediate hosts [1].

Among meat-producing animals, cattle (*Bos taurus*) are common intermediate hosts of *Sarcocystis* spp., whose prevalence in muscle can reach up to 100% [2]. Although there has recently been confusion about the validity and classification of several *Sarcocystis* spp. from cattle, it is now generally agreed that bovine muscle tissue can harbor at least six *Sarcocystis* spp., the well-known *S. cruzi*, *S. hirsuta* and *S. hominis*, with felids, canids and humans, respectively, as definitive hosts, and the recently added *S. bovifelis*, *S. bovini* and *S. heydorni*, with felids acting as definitive hosts for the first two species and primates acting as definitive hosts for the latter species [3–5].

The consumption of raw or undercooked beef meat constitutes an important risk factor for humans, who become infected by ingesting muscular sarcocysts [6]. Symptoms of intestinal sarcocystosis, such as nausea, abdominal pain and diarrhea, can have a wide range of intensity, depending on the number of ingested cysts and on the immune response of the host, though most infections go unnoticed [6]. In addition to the zoonotic potential, there is increasing interest around these protozoa in the food industry due to the evidence of their association with bovine eosinophilic myositis (BEM), a specific inflammatory myopathy with multifocal gray-green lesions which leads to carcass condemnation and considerable economic losses [7]. Worldwide, BEM reported prevalence in slaughtered cattle ranges from 0.002 to 5% [8]. These data might appear inconsistent with the high prevalence of *Sarcocystis* in cattle; in this regard, little evidence has pointed out as possible explanation that BEM might be associated with one or more *Sarcocystis* species [8].

Prevalence data reported from cattle in Italy are consistent with European reports, revealing a *Sarcocystis* spp. prevalence of 96% [9], 80% [10], 91% [11] and 88% [12]. All these studies have been based on morphological techniques or on molecular techniques targeting the nuclear small subunit (18S) rDNA gene; however, the suitability of this locus for distinguishing between closely related *Sarcocystis* spp. has recently been challenged [13]. Indeed, though public databases contain mostly 18S rDNA sequences because of its high use for *Sarcocystis* identification, cytochrome C oxidase subunit I mitochondrial (mtDNA *cox1*) gene is actually seen as the most promising tool to differentiate closely related *Sarcocystis* spp. using ungulates as intermediate hosts [3, 14]. In particular, as highlighted by Moré et al. [15], sequence differences among *S. hominis*, *S. bovifelis* and *S. bovini* are approximately 3% of the 18S rDNA gene; therefore, using only size differences of the amplified 18S rDNA fragments may result in the misidentification of these species [2, 15].

In the light of this, data resulting from the prevalence studies carried out in Italy in the last years might have led to an overestimation of *S. hominis* prevalence [16], apparently ranging from 42.7% [10] to 68% [12].

Thus, the aim of the present study was to evaluate the prevalence of the different *Sarcocystis* spp. in slaughter cattle from Northwest Italy and in BEM condemned carcasses, focusing on the hypothesis that BEM might be associated with specific *Sarcocystis* spp. [8, 11].

## Materials and methods

### Sample collection and processing

**Fig. 1:**
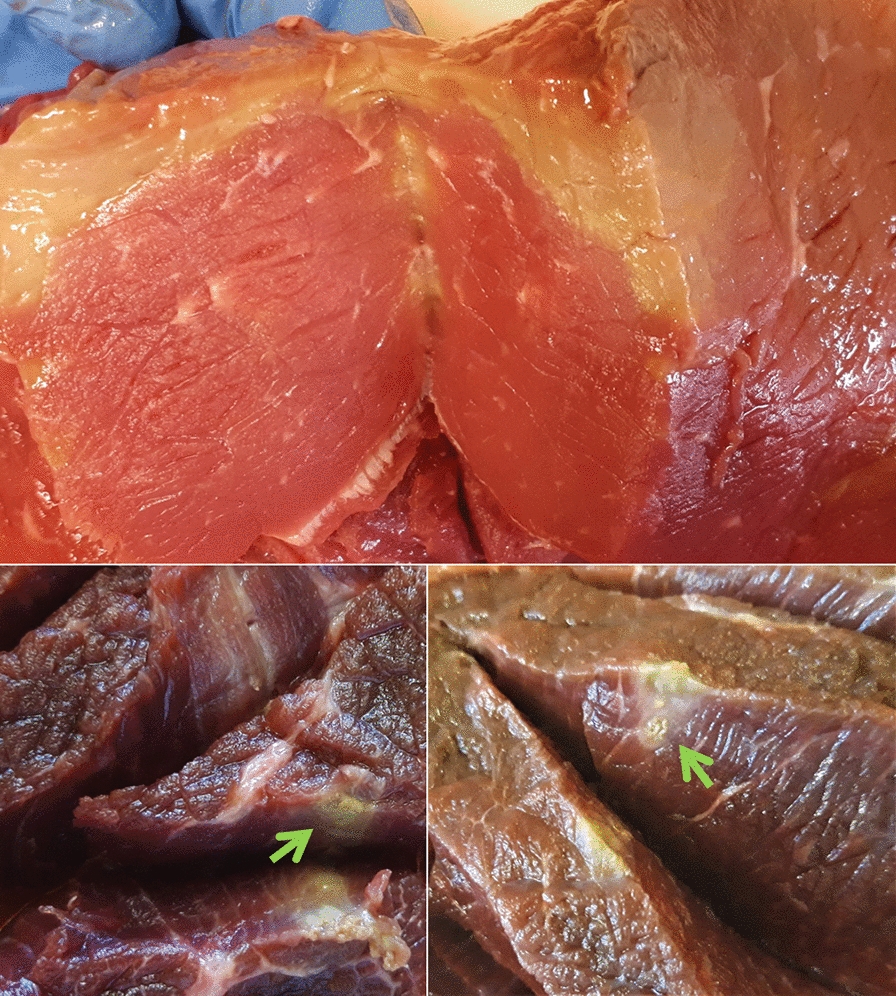
Muscle samples with cut sections showing the presence of typical focal and diffuse gray-green lesions; central cores of suppuration are marked by the green arrows

From January 2012 to July 2020, striated muscle samples from 54 BEM condemned carcasses were submitted by different slaughterhouses located in Northwest Italy to the Laboratory of Food Inspection at the Department of Veterinary Sciences (University of Turin, IT) for etiological confirmation. Muscle samples were macroscopically examined for the presence of typical focal or diffuse gray-green lesions (Fig. 1); detected lesions were excised and stored at − 20 °C for further analysis. Simultaneously, in 2019–2020 the diaphragm muscles of 59 slaughter cattle were collected from Piedmont (Northwest Italy) slaughterhouses, for a total of 113 individual cattle samples. Tissue samples were collected by veterinarians during post-mortem inspections of slaughtered animals and then transported to the laboratory at refrigeration temperature; 25 mg of tissue for each individual muscle sample was collected and stored at − 20 °C until further analysis.

### DNA extraction and molecular detection of *Sarcocystis* spp.

DNA extraction was performed using DNeasy Blood and Tissue Kit (Qiagen, Hilden, Germany), according to the manufacturer’s tissue protocol; the lysis step was carried out at 56 °C overnight with Proteinase K. DNA samples were eluted in 50 μl of elution buffer and kept frozen at − 20 °C. The identification of different *Sarcocystis* spp. was performed through the application of the multiplex-PCR assay described by Rubiola et al. [16] targeting the 18S rDNA gene and the mtDNA *cox1* gene. The multiplex-PCR contained 2.5 μl of template DNA (5–20 ng/μl), 0.5 mM of each primer, Sarco Rev, Sar F, Hirsuta, Cruzi, COI HB, COI H and COI B, 2 mM MgCl2, 0.2 mM of each dNTP, 1 U Platinum Taq DNA polymerase, 10 × PCR buffer and RNase-free water to a total volume of 25 μl. The amplification was performed in an Applied Biosystems 2720 Thermal Cycler (AppliedBiosystems, CA, USA) with the following cycling profile: a denaturation step at 95 °C for 3 min, followed by 35 cycles at 95 °C for 60 s, 58 °C for 60 s and 72 °C for 30 s and final extension 72 °C for 3 min. In each PCR run, 2.5 μl of DNA from a collection of *Sarcocystis*-positive samples isolated from cattle striated muscle in the Department of Veterinary Science of Turin University [11, 16, 17] were used as positive controls while extracted DNA from negative cattle muscles as well as reagent blanks were included as negative controls. PCR products were observed in 2% agarose gel stained with SYBR safe stain (Invitrogen, Carlsbad, CA) and observed in a blue light transilluminator (Invitrogen, Groningen, The Netherlands).

### Sanger sequencing and phylogenetic analysis

PCR products amplified through the use of the genus-specific primer set in the absence of the specific fragment for *S. hirsuta*, *S. cruzi*, *S. hominis* or *S. bovifelis* were sequenced to achieve species identification. PCR amplicons were purified with Exo-Sap treatment (USB Europe, Staufen, Germany) according to the manufacturer's instructions. Forward and reverse sequencing reactions were performed using ABI Prism BigDye Terminator Cycle Sequencing Ready Reaction Kit, version 1.1 (Applied Biosystems, Foster City, CA). Sequenced fragments were purified by DyeEX (Qiagen, Hilden, Germany), and sequence analysis was performed on an Applied Biosystems 310 Genetic Analyzer (Applied Biosystems, Foster City, CA). The nucleotide sequences were analyzed using the BLASTN sequence similarity search at the NCBI database [18]. Phylogenetic analyses of the 18S rDNA gene sequences were performed using the neighbor-joining method [19] within MEGA7 [20]. *Sarcocystis* spp. reference sequences included are shown in Additional file 1.

### Statistical analysis

Fisher’s exact test was used to compare the proportions and 95% confidence intervals (CIs) of different *Sarcocystis* spp. in BEM condemned carcasses and in slaughter cattle carcasses. *P* ≤ 0.05 was considered significant.

## Results

### Prevalence of *Sarcocystis* spp. in cattle carcasses

**Fig. 2:**
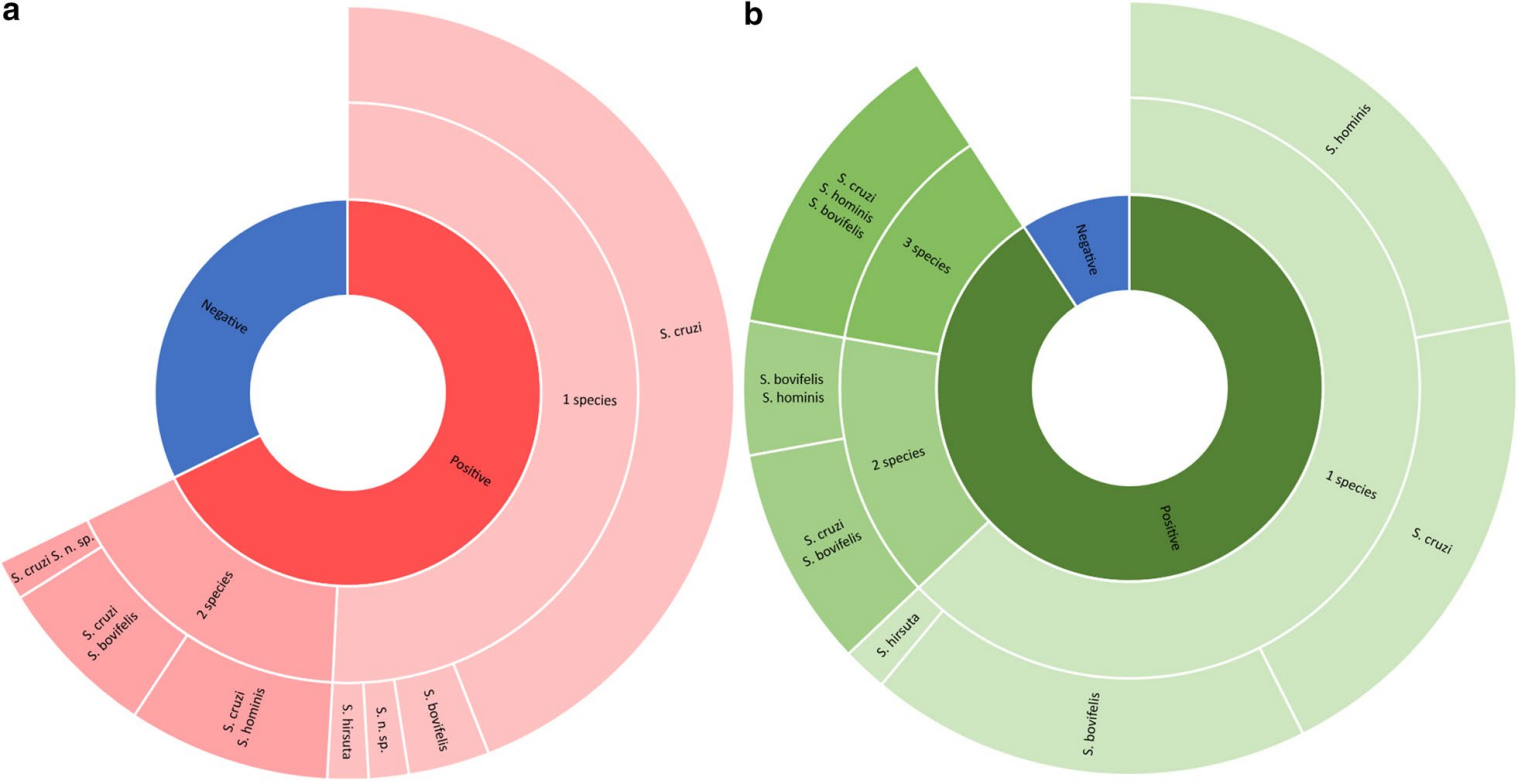
Sunburst charts showing the distribution of different *Sarcocystis* spp. and co-infestations in slaughter cattle carcasses (**a**) and BEM condemned carcasses (**b**) analyzed in this study

Out of 59 individual samples from randomly sampled cattle carcasses, *Sarcocystis* DNA was detected in 67.8% (40/59; 95% CI 55.06–78.36%) of the muscle samples. *S. cruzi* was the most common species (61%, 36/59; 95% CI 48.25–72.44%), while *S. bovifelis*, *S. hominis*, *S. hirsuta* and an unidentified *Sarcocystis* sp. counted for 10.2% (6/59; 95% CI 4.40–20.81%), 8.5% (5/59; 95% CI 3.27–18.75%), 1.7% (1/59; 95% CI < 0.01–9.85%) and 3.4% (2/59; 95% CI 0.26–12.22%), respectively (Fig. 2a). Mixed infections were observed in 16.9% (*n* = 10) of the samples, revealing the presence of up to two species of *Sarcocystis* at once. In cases of a single species being detected (*n* = 30), *S. cruzi* was the most common finding (86.7%, *n* = 26), followed by *S. bovifelis* (6.7%, *n* = 2), *S. hirsuta* (3.3%, *n* = 1) and an unidentified *Sarcocystis* sp. (3.3%, *n* = 1). When two *Sarcocystis* spp. were detected in the same sample (*n* = 10), the simultaneous presence of *S. cruzi* and *S. hominis* was the most common finding (50%), followed by co-infection of *S. cruzi* and *S. bovifelis* (40%) and of *S. cruzi* and the unidentified *Sarcocystis* sp. (10%), while no muscle samples revealed the simultaneous presence of three or more *Sarcocystis* spp.

### Prevalence of *Sarcocystis* spp. in BEM condemned carcasses

Out of 54 individual samples from BEM condemned carcasses, *Sarcocystis* DNA was detected in 90.7% (49/54; 95% CI: 79.67–96.40%) of the muscle samples. The majority of intralesional *Sarcocystis* spp. were found to be *S. bovifelis* (46.3%, 25/54, 95% CI 33.69–59.40%), followed by *S. cruzi* (42.6%, 23/54; 95% CI 30.32–55.85%) and *S. hominis* (40.7%, 22/54, 95% CI 28.66–54.05%), while *S. hirsuta* counted for 1.8% (2/54; 95% CI 0.30–13.26%) (Fig. 2b). Mixed infections were observed in 27.8% (*n* = 15) of the samples, revealing the presence of up to three species of *Sarcocystis* at once. Among co-infestations, the most common finding was the presence of two species (53.3%, *n* = 8), while three species were detected in seven samples (46.7%). In case of a single species being detected (*n* = 34), *S. hominis* was the most common finding (35.3%, *n* = 12), followed by *S. cruzi* (32.4%, *n* = 11), *S. bovifelis* (29.4%, *n* = 10) and *S. hirsuta* (2.9%, *n* = 1). When two *Sarcocystis* spp. were detected in the same sample, the simultaneous presence of *S. cruzi* and *S. bovifelis* was the most common finding (62.5%), followed by co-infection of *S. bovifelis* and *S. hominis* (37.5%), while all muscle samples harboring three species revealed the simultaneous presence of *S. cruzi*, *S. bovifelis* and *S. hominis*.

### Sanger sequencing and phylogenetic analysis

**Fig. 3:**
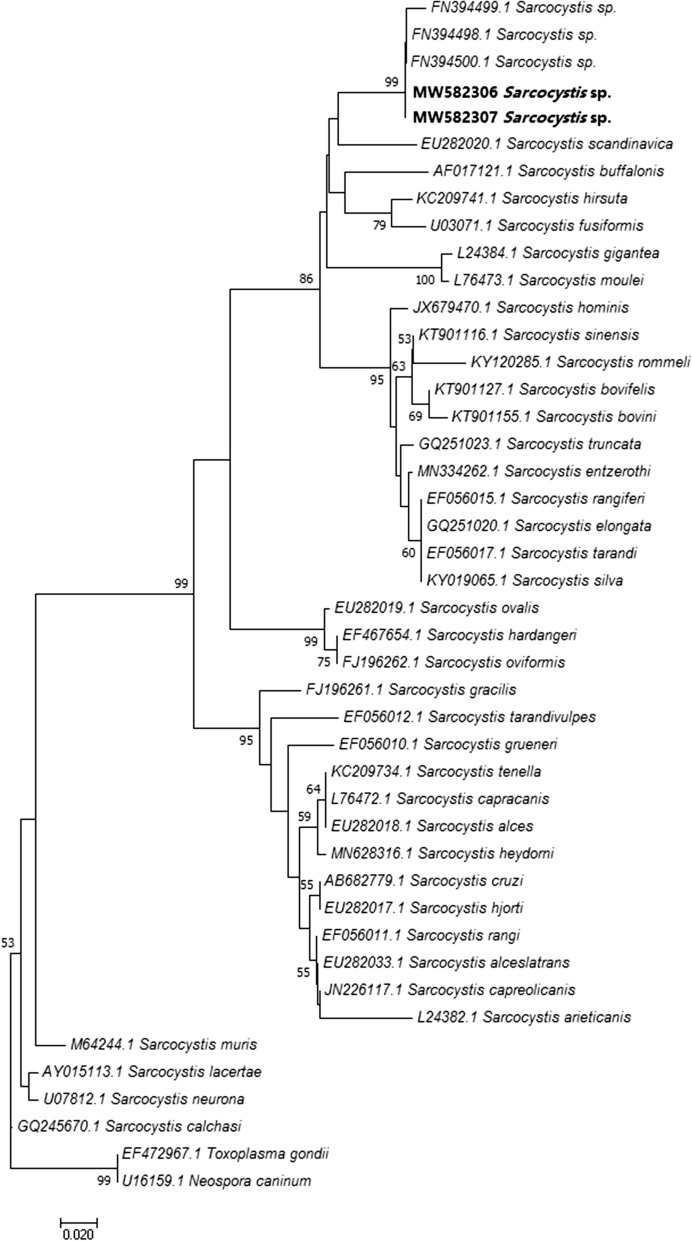
Neighbor-joining phylogenetic tree for members of the *Sarcocystidae* based on 18S rDNA sequences of 37 *Sarcocystis* spp. and including the unidentified *Sarcocystis* spp. sequences isolated in this study (in bold) and three GenBank entries (Accession Nos. FN394500.1, FN394498.1, FN394499.1) corresponding to unidentified *Sarcocystis* spp. [8]. *Toxoplasma gondii* and *Neospora caninum* were used as outgroups. The percentage of replicate trees in which the associated taxa clustered together in the bootstrap test (1000 replicates) are shown next to the branches. Bootstrap values < 50 are not shown

Two out of 59 individual samples from randomly sampled cattle carcasses revealed the presence of a PCR product amplified through the use of the genus specific primer set, in the absence of the specific fragment for *S. hirsuta*, *S. cruzi*, *S. hominis* or *S. bovifelis*. Consensus sequences of the 18S rDNA fragments were 152 bp in length and showed < 86.43% similarity to any known *Sarcocystis* spp. sequence deposited in GenBank. Notably, these amplicons showed a sequence homology ≥ 95.42–99.34% with three GenBank entries (accession no. FN394498—FN394500) corresponding to unidentified *Sarcocystis* spp. isolated from cattle muscle samples [8]. The obtained 18S rDNA sequences were deposited in Genbank under accession no. MW582306, MW582307. A phylogenetic analysis on the unidentified 18S rDNA sequences and on representative sequences deposited in GenBank was inferred using the neighbor-joining method [19] within MEGA7 [20]; the resulting phylogenetic tree is shown in Fig. 3. The unidentified 18S rDNA sequences from this study formed a monophyletic cluster together with GenBank entries FN394498—FN394500 within the clade including cat-transmitted *Sarcocystis* spp. with ruminant intermediate hosts.

### Statistical analysis

The presence of *Sarcocystis* spp. DNA was significantly higher in samples isolated from BEM condemned carcasses than in samples isolated from randomly sampled cattle carcasses (Fisher’s exact test two-tailed, *P* = 0.0050). Among the different *Sarcocystis* spp. detected, the presence of *S. bovifelis* or *S. hominis* was significantly higher in samples isolated from BEM condemned carcasses than in samples isolated from randomly sampled cattle carcasses (Fisher’s exact test two-tailed, *P* < 0.0001), while there was no statistically significant difference between the presence of *S. cruzi*, *S. hirsuta* or the unidentified *Sarcocystis* sp. in BEM condemned carcasses and randomly sampled carcasses (Fisher’s exact test two-tailed, *P* = 0.0606, *P* > 0,9999, *P* = 0,4965, respectively).

## Discussion

Cattle sarcocystosis is gaining importance as one of the causes of bovine eosinophilic myositis, a specific inflammatory myopathy which leads to serious economic outcomes in the beef sector [2, 11]. Thus, species identification of intra-lesional *Sarcocystis* is crucial to better understand the contribution of specific species to BEM pathogenesis and to explain the low prevalence of BEM lesions [16], despite the high prevalence of sarcocysts in the cattle population. Therefore, the aim of this study was to evaluate the presence of *Sarcocystis* spp. in Italian slaughter cattle and in BEM condemned carcasses to update the prevalence data reported from cattle in Italy in light of the recent taxonomic revision of cattle *Sarcocystis* and to evaluate the hypothesis that BEM might be associated with specific *Sarcocystis* spp. [8].

In our study, the 67.8% prevalence of *Sarcocystis* spp. in diaphragm samples randomly taken from Northwest Italy slaughterhouses (*n* = 59) is compatible with the high prevalence previously reported [9–12]. *S. cruzi* has been confirmed as the most common species, followed by *S. bovifelis* and *S. hominis*, while *S. hirsuta* DNA was only detected in 1.7% of samples. Notably, the 8.5% prevalence of *S. hominis* here detected is much lower than previously reported (43–68%), while *S. bovifelis* has never been considered in prevalence studies carried out in Italy so far [9–12], since at that time it still had to be described [21]. These findings highlight the previous overestimation of *S. hominis* prevalence due to the detection techniques based on the lower discriminative 18S rDNA gene; this evidence suggests that *S. bovifelis* might have been misidentified as *S. hominis*, thus explaining its absence in all previous prevalence studies carried out in Italy [16]. Furthermore, the examined striated and diaphragm muscle samples have proved to be suitable matrices to investigate the presence of different *Sarcocystis* spp. This tendency has been previously confirmed in several studies [10, 22, 23] examining different tissue (e.g. heart, skeletal muscle, esophagus, diaphragm, tongue) and confirmed by others processing only heart muscle samples [24, 25]. Therefore, the tissue matrices can considerably influence the detection of different *Sarcocystis* spp.

Considering the high detection of *S. cruzi*, followed by *S. bovifelis* and *S. hominis*, the prevalence data reported in our study show most resemblance to those of Hungary [22] and The Netherlands [26], while in Germany and Lithuania a higher prevalence of *S. hirsuta* is reported, ranging from 6.6 to 30.4%, respectively [27, 28].

The presence of *Sarcocystis* spp. in BEM condemned carcasses differed significantly with respect to the previously described group of randomly sampled slaughter cattle. In particular, the detection of *Sarcocystis* spp. DNA in 90.7% of the BEM condemned carcasses was significantly higher than in unaffected cattle. This finding confirms the association of *Sarcocystis* spp. with BEM lesions, though the presence of *Sarcocystis* spp. is not exclusively associated with lesions typical for bovine eosinophilic myositis [26]. Besides, among the different *Sarcocystis* spp. detected, the presence of *S. bovifelis* and *S. hominis* was significantly higher in samples isolated from BEM condemned carcasses than in samples isolated from randomly sampled slaughter cattle. This finding supports the hypothesis that BEM might be associated with specific *Sarcocystis* spp. Literature on this topic is confusing since several species have been reported in association with eosinophilic lesions [7, 8, 26, 29–32]; among these studies, both thin- and thick-walled *Sarcocystis* spp. are reported, including *S. hominis* [7, 8, 26, 29] and *S. cruzi* [32]. However, most of these reports have been based on morphological identification, which cannot discriminate among closely related *Sarcocystis* spp.; besides, this method is affected by the damage of the cyst walls which is often present in BEM lesions [8]. Molecular detection techniques were applied by Vangeel et al. [8], and the majority of intralesional *Sarcocystis* were found to be *S. hominis*, though also *S. cruzi* and *S. hirsuta* were found in BEM lesions; however, *S. bovifelis* was not recognized as a different *Sarcocystis* spp. until its resurrection in 2016 [21]. Our findings lead us to hypothesize a major role for *S. hominis* and *S. bovifelis* in bovine eosinophilic myositis, though also *S. cruzi* was detected in BEM condemned carcasses. To corroborate our current findings, as well as to update knowledge on this disease, which leads to serious economic outcomes in the beef sector, further research is required. In particular, multiple sampling in BEM-affected carcasses, involving both intralesional and extralesional tissue, might be performed to evaluate the presence of different *Sarcocystis* spp. outside and inside lesions.

In the present study, an unidentified species was detected in two carcasses; although the small size of the sequenced fragment cannot give consistent molecular results, as highlighted by the low bootstrap values reported in the phylogenetic tree (Fig. 3), this species seems to be most closely related to thick-walled *Sarcocystis* spp. from bovids and cervids. Interestingly, as suggested by the high percentage of identity reported in the phylogenetic analysis (Fig. 3), a 177-bp 18S rDNA fragment of this unidentified species has already been sequenced in association with BEM lesions in Belgium [8], though no further research was performed at that time. Further investigations are needed to characterize this putative new species and investigate its cycle and possible role in BEM pathogenesis.

The detection of the zoonotic *S. hominis* confirms the established transmission cycle between cattle and humans in Italy, pointing out the risk for the consumer of raw or undercooked beef. Human intestinal sarcocystosis is well documented in the literature, in both asymptomatic patients and patients with gastrointestinal symptoms [6]. Recently, the presence of *S. hominis* in six patients hospitalized with gastrointestinal symptoms has been reported in the Piedmont region, Northwest Italy, which is well known for raw beef consumption [16]. Therefore, epidemiological data on this and other actually undetected zoonotic species must be considered of importance from a public health perspective.

## Conclusions

In conclusion, the results of our study contribute to updating the data on the prevalence of the different *Sarcocystis* spp. from cattle in Italy, highlighting the previous overestimation of *S. hominis* due to the use of morphological methods or ineffective 18S rDNA-based molecular techniques. Besides, our findings contribute to the understanding of the importance of different *Sarcocystis* spp. in BEM pathogenesis, highlighting the presence of three species, *S. cruzi*, *S. hominis* and *S. bovifelis*, in BEM lesions and allowing us to speculate on the possible role of *S. hominis* and *S. bovifelis* as the major sarcosporidian species involved. Lastly, considering the detection of the zoonotic *S. hominis*, the results of the current survey highlight a substantial public health concern and offer useful information for public health specialists.

## Supplementary Information

**Additional file 1: Table S1.** Reference sequences downloaded from GenBank and used in this study.

## Data Availability

All datasets generated for this study are included in article/additional files. The sequences generated in the present study are available in GenBank database with Accession Numbers MW582306, MW582307.
