# Supplementary material for: Molecular detection of cattle Sarcocystis spp. in North-West Italy highlights their association with bovine eosinophilic myositis
Source: Parasit Vectors. 2021 Apr 23;14:223. doi: 10.1186/s13071-021-04722-5 (PMC8063337; doi:10.1186/s13071-021-04722-5)
Supplement: Supplementary file 1 — Additional file 1: Table S1. Reference sequences downloaded from GenBank and used in this study. [file 13071_2021_4722_MOESM1_ESM.docx]

**Additional file 1.** Reference sequences downloaded from GenBank and used in this study.

| **Species** | **Strain** | **18S rDNA** |
| --- | --- | --- |
| *Sarcocystis* sp.* | Ssp 14 | FN394499.1 |
| *Sarcocystis* sp.* | Ssp 13 | FN394498.1 |
| *Sarcocystis* sp.* | Ssp 15 | FN394500.1 |
| *S. Scandinavica* | SsAa1N clone 1.1 | EU282020.1 |
| *S. buffalonis* | / | AF017121.1 |
| *S. hirsuta* | B 5.1 | KC209741.1 |
| *S. fusiformis* | / | U03071.1 |
| *S. gigantea* | / | L24384.1 |
| *S. moulei* | / | L76473.1 |
| *S. hominis* | Clone 1B HRF93A | JX679470.1 |
| *S. sinensis* | Bb21.27 | KT901116.1 |
| *S. rommeli* | Srs2B | KY120285.1 |
| *S. bovifelis* | B4.1 clone 4 | KT901127.1 |
| *S. bovini* | B7.2 clone 6 | KT901155.1 |
| *S. truncata* | Srf1CeN clone 3 | GQ251023.1 |
| *S. entzerothi* | CcLT24.3a | MN334262.1 |
| *S. rangiferi* | Srf1RtN | EF056015.1 |
| *S. elongata* | St2CeN clone 5 | GQ251020.1 |
| *S. tarandi* | St1RtN | EF056017.1 |
| *S. silva* | Ccl4.50 clone 4 | KY019065.1 |
| *S. ovalis* | SoAa1N | EU282019.1 |
| *S. hardangeri* | ShRt1l | EF467654.1 |
| *S. oviformis* | / | FJ196262.1 |
| *S. gracilis* | / | FJ196261.1 |
| *S. tarandivulpes* | Stv1RtN | EF056012.1 |
| *S. grueneri* | Sg1RtN | EF056010.1 |
| *S. tenella* | S2.1 | KC209734.1 |
| *S. capracanis* | / | L76472.1 |
| *S. alces* | SaAa1N | EU282018.1 |
| *S. heydorni* | QAQHA-1 | MN628316.1 |
| *S. cruzi* | NSMT: Pr316 | AB682779.1 |
| *S. hjorti* | SspEAa1N | EU282017.1 |
| *S. rangi* | Sr1RtN | EF056011.1 |
| *S. alceslatrans* | SalAa1C | EU282033.1 |
| *S. capreolicanis* | NoCc10.7 clone 1 | JN226117.1 |
| *S. arieticanis* | / | L24382.1 |
| *S. muris* | / | M64244.1 |
| *S. lacertae* | / | AY015113.1 |
| *S. neurona* | SN5 | U07812.1 |
| *S. calchasi* | / | GQ245670.1 |
| *Toxoplasma gondii* | RH | EF472967.1 |
| *Neospora caninum* | N.C.-Liverpool | U16159.1 |

*Unidentified *Sarcocystis* sp.
